# Letter: The difference and clinical application of modified thoracolumbar fracture classification scoring system in guiding clinical treatment

**DOI:** 10.1186/s13018-023-04343-x

**Published:** 2023-11-20

**Authors:** Mohamed M. Aly

**Affiliations:** 1 Department of Neurosurgery, Prince Mohammed Bin Abdulaziz Hospital, P.O Box 54146, 11514 Riyadh, Saudi Arabia; 2 Department of Neurosurgery, Mansoura University, Mansoura, Egypt

Letter to Editor

We read with great interest the paper by Wenjie Li et al. entitled: The difference and clinical application of modified thoracolumbar fracture classification scoring system in guiding clinical treatment [1]. The authors retrospectively compared the correlation of the Thoracolumbar injury classification score (TLICS) and the newly proposed modified TLICS with treatment recommendations in 120 patients with thoracolumbar. The study revealed no statistically significant difference in the total score or treatment method between the TLICS and modified TLICS systems. However, the modified TLICS system's operation rate was slightly lower than the TLICS system (73.3 vs.79.2%) [1]. While recognizing the authors' efforts in this study, we have a few remarks to add.

The modified TLICS classification attempts to improve the decision-making for burst fractures in neurologically intact patients with or without indeterminate PLC status, often in the *''grey zone''*. Nevertheless, the modified TLICS does not account for the severity of burst fractures, whereas the AOSpine thoracolumbar injury severity score (TLAOSIS) assigns more points to A4 fractures than A3 fractures (five vs. three, respectively) [2]. We agree that high signal intensity, indicative of "indeterminate PLC integrity," is assigned a score of 1 by modified TLICS since it contributes so little to PLC incompetence [3–5]. Nonetheless, if a complete PLC injury is only worth 2 points, then all burst fractures with complete PLC injury will be in the *''grey zone''*. This contradicts the widely held belief that complete PLC damage should be addressed surgically to avoid delayed instability and kyphosis. [6]

The "intervertebral disc injury status" evaluation in modified TLICS uses the Sander classification and scores 0, 1, and 2 points for no injury, mild injury, and moderate-to-severe injury [7]. Again, you would expect the intervertebral disc injury to have the highest impact on burst fractures in neurologically intact patients. However, it is unclear from the data presented how the severity of disc injury modified the treatment recommendation for that group of patients. Overall, the number of patients in the grey zone has remained constant, while the number of patients receiving surgical recommendations has decreased somewhat in favour of conservative. This implies that the severity of disc injury has little impact on decision-making. However, the authors should have presented detailed data for modified TLICS vs. TLICS score for burst fractures to display the impact of intervertebral disc injury vs. reducing PLC score in that group.

Given the limited availability of MRI worldwide, both TLICS and AOSpine categorization relies on the morphology of fractures on computed tomography (CT) [8, 9]. Relying on MRI for modified TLICS will reduce the applicability of the classification. MRI is unquestionably the only technique to assess PLC and disc damage directly. In that context, recent studies have proposed a new criterion to predict the probability of PLC injury in MRI. We have shown that the following four CT findings are independent predictors for PLC injury: spinous process fracture, horizontal laminar fracture, facet diastasis, and interspinous widening > 4 mm. [10] They proposed the following definition of PLC status in CT: PLC injury ≥ 2 CT findings: PPV for PLC injury is 91%, suspected PLC injury (AKA M1 modifier) PPV 33%, and intact PLC no positive CT findings PPV 9% [10]. IF those findings are externally validated, it could help improve the CT accuracy of PLC injury. Similarly, it would be intriguing to look for CT surrogate markers for disc injuries in order to incorporate them into CT-based classifications.

## Data Availability

Not applicable.
